# Maternal Perception vs Actual Breast Milk Supply: Protocol for an Observational Cross-Sectional Study

**DOI:** 10.2196/84776

**Published:** 2026-03-31

**Authors:** Bailey Bruckner, Rachael Taylor, Carmen Parata, Jillian Haszard, Barry Taylor, Samantha Bevin, Kassidy Gooding, Anne-Louise Heath, Ioanna Katiforis, Lisa Daniels

**Affiliations:** 1 Department of Human Nutrition University of Otago Dunedin New Zealand; 2 Department of Medicine University of Otago Dunedin New Zealand; 3 Department of Public Health University of Otago Wellington New Zealand; 4 Haszard Biostatistics Otago New Zealand; 5 Department of Paediatrics and Child Health University of Otago Dunedin New Zealand; 6 School of Exercise and Nutrition Sciences Deakin University Burwood Australia

**Keywords:** breastfeeding, human milk composition, human milk, infants, lactation, milk supply, perceived insufficient milk supply, PIMS, stable isotope

## Abstract

**Background:**

It is well known that breastfeeding provides favorable health outcomes for both mother and baby. However, many mothers struggle to meet global recommendations to exclusively breastfeed for 6 months. Of those who cease breastfeeding early, one third attribute this to perceived insufficient milk supply. Currently, it is uncertain how the perception of insufficient milk supply relates to physiological milk volume or nutrient composition.

**Objective:**

The Māmā and Baby Breastfeeding Study aims to estimate human milk volumes produced by a diverse sample of breastfeeding mothers at 3 months post partum with differing perceptions of milk supply and to investigate human milk composition in relation to milk volume.

**Methods:**

We plan to recruit a sample of 150 mother–infant dyads in this observational study in Dunedin, Aotearoa New Zealand. Participants will represent a diverse range of ethnicities and socioeconomic backgrounds. Human milk volume will be assessed using the dose-to-mother stable isotope (deuterium oxide) technique. Mother participants will consume an accurate dose (30 g) of deuterium oxide after baseline saliva samples are collected from both mother and infant. Subsequent postdose samples will be collected over 3 time points to determine deuterium enrichment over a 14-day period using Fourier transform infrared spectrometry. Human milk macronutrient (energy, fat, carbohydrate, crude protein, and true protein) and mineral and trace element (sodium, magnesium, phosphorus, potassium, calcium, iron, copper, zinc, selenium, and iodine) composition of 1 full milk expression from 1 breast will be analyzed using the MIRIS Human Milk Analyzer (Miris AB) and inductively coupled plasma mass spectrometry, respectively. Potential predictors and maternal perception of milk supply will be assessed via questionnaire. Infant BMI will be calculated from measures of weight and length at 3 different time points over 4 weeks, using standard techniques. These, alongside anthropometric measurements collected at the infant’s Well Child Tamariki Ora visits, will be used to assess infant growth trajectory in the first 6 months of life. Regression models will be used to assess the associations between maternal perception of milk supply, human milk volumes, and composition.

**Results:**

This study was funded in April 2023 by the Health Research Council of New Zealand (grant 23/461). Recruitment for this study began in February 2025 and is anticipated to conclude in June 2026, with analysis expected to be completed by February 2027. As of January 17, 2026, a total of 91 participants have been enrolled. Final results are anticipated to be disseminated in late 2027 following completion of data analysis.

**Conclusions:**

This research will provide new knowledge on whether maternal perception of milk supply aligns with actual human milk volume or nutrient composition. Such information will be extremely useful for health professionals working with breastfeeding mothers with milk supply concerns and for informing the design of breastfeeding support programs and resources.

**Trial Registration:**

Australian and New Zealand Clinical Trial Registry ACTRN12625000180415; https://www.anzctr.org.au/ACTRN12625000180415.aspx

**International Registered Report Identifier (IRRID):**

DERR1-10.2196/84776

## Introduction

### Overview

While national and international advisory groups recommend exclusive breastfeeding for the first 6 months of life and continued breastfeeding for children aged 2 years or older [1,2], many mothers in New Zealand and globally are not meeting these recommendations. In New Zealand, 77% of infants are exclusively breastfed at discharge from maternity services [3]; however, this drops to under 60% by 4 months of age and less than 10% by 6 months of age [4]. These percentages are lower than exclusive breastfeeding rates at 6 months in the United States (24.9% in 2019) [5] but higher than recent estimates in Australia (less than 1% in 2021) [6]. It is evident that work is needed to improve exclusive breastfeeding rates for the first 6 months of life in New Zealand and other high-income countries, especially considering the global goal of 60% of infants being exclusively breastfed to 6 months of age by 2030 set by the World Health Assembly [7].

There are many reasons why mothers may not meet recommended breastfeeding targets, such as pain, tiredness, inconvenience, or return to work or education [8,9]. However, perceived insufficient milk supply (PIMS)—a mother’s perception that her production of milk is not adequate to satisfy her infant’s needs—is reported to be the leading cause of early cessation of breastfeeding [10-12]. Gatti [10] concluded that, of women who discontinue breastfeeding early, 35% describe PIMS as the primary reason. It is also a known barrier to continuing breastfeeding in New Zealand [13,14]. However, while PIMS is the most commonly cited reason for early cessation of breastfeeding, it is not currently known how many experience PIMS but continue to breastfeed despite these challenges.

Factors that commonly contribute to this perception often include unsettled infant behaviors, such as frequent crying or short sleep duration, as well as low maternal satisfaction and self-confidence in breastfeeding [15]. However, the perception of insufficient milk supply can be a dangerous cycle that can lead to actual low milk supply through disruption of the supply-demand feedback loop [16]. If supplementation with other milks, such as infant formula, occurs due to the perception of low milk supply, this can cause a decrease in feeding at the breast and therefore a reduction in milk production [17,18]. Maternal feelings of personal failure, guilt, anxiety, and reduced maternal self-efficacy can occur as a consequence of perceived breastfeeding failure [9,19], potentially exacerbating the symptoms of PIMS.

A major gap in the literature exists in that, while PIMS is very common and leads to a large proportion of early breastfeeding cessation, how perception of insufficient milk supply relates to actual physiological milk volume is uncertain. Similarly, there appears to be no research investigating the perception of high milk volume (oversupply) in this context. Only 1 study appears to have investigated the relationship between PIMS and human milk volume [20], using the 24-hour test-weighing method in first-time mothers only. The study found that, at 2 weeks post partum, there was no significant association between perceived and actual insufficient milk supply. No other research has examined older infants, particularly infants aged 3 months, immediately before breastfeeding rates decrease in New Zealand [4]. This is perhaps due in part to the fact that accurately measuring human milk intake is complex. Standard approaches often assume a fixed volume of intake across infants of a specific age. For example, a commonly used “one size fits all” approach estimates individual intake at 780 mL/day for infants aged 0 to 6 months [21]. However, such estimates mask considerable variation observed between mother-infant pairs, with intakes ranging from 400 to 1400 mL/day in infants aged 2 to 5 months [22]. Such standard approaches clearly undermine the ability to assess true intake in breastfed infants.

Other approaches include 24-hour test-weighing, in which the mother weighs the infant directly before and after all breastfeeds over a 24-hour period. Test-weighing, while more individualized than the “one size fits all” approach, may impact usual feeding patterns and interfere with the physiological and behavioral aspects of lactation [23,24]. Measuring pumped (expressed) milk volume has also been used to estimate human milk production [25-27]. However, this is not considered an accurate indicator of infant intake largely due to differences between maternal production capacity and infant need [27]. Pumping output is also highly variable, influenced by factors such as device efficiency, maternal pumping technique, and individual responsiveness to pumping [28,29]. As a result, expressed volume often reflects the effectiveness of the tools and methods used rather than the mother’s underlying physiological milk production.

Another common method of determining human milk intake is the “minutes per feed” approach, which assumes that a set amount of milk is transferred to the baby per minute of breastfeeding. However, ultrasound imagery has previously found no relationship between the length of the breastfeed and the amount of milk transferred [30], suggesting considerable inaccuracy with this method. A promising approach to estimating human milk intake is the use of predictive modeling [31]; although, to date, only limited data are available and the method has yet to be validated.

Finally, the dose-to-mother (DTM) stable isotope technique [24,32,33] is considered the gold-standard method for estimating human milk volume. This method can estimate human milk intake over a 14-day period by giving the mother an accurate oral dose of deuterium oxide (D_2_0; a stable isotope, also known as “heavy water”) and tracing the transfer of D_2_O from the mother to the infant through mother and infant saliva or urine samples. This method has been used to estimate human milk intake of infants worldwide, most commonly in early infancy [23,34-38], but also in older infants aged 7 to 10 months in New Zealand [31]. While it is more expensive than other methods, it is relatively noninvasive and provides an accurate estimate of human milk intake [33]. To date, no research has used the gold-standard DTM technique to investigate the relationship between PIMS and actual milk intake in infants.

To add to the complexity of measuring human milk intake, infant intake can be influenced by a range of factors. Well-known maternal predictors of milk intake include higher education, which is associated with higher intakes [31,39], as well as current employment and high BMI, both associated with lower intakes [31,40]. Well-known infant predictors include age [23,31,39,41,42], with intake varying over the course of lactation, as well as high body weight [22,31,34,39,43], demand vs scheduled feeding [31,44], and a higher average number of breastfeeds [31,45], which are associated with higher intake in infants. However, many factors that may impact infant milk intake have not been well investigated. For example, maternal cannabis or nicotine use has been suggested to be associated with lower infant milk intake [46,47], as well as certain maternal medications [48], poor maternal mental health [49], high maternal stress [50], and infant male sex [23], among others. These potential predictors have not been thoroughly investigated, and further exploration is needed to understand their impact.

It is also not known how differences in nutritional composition influence the perception of milk supply or volume. Determining human milk adequacy in terms of both volume (quantity) and composition (quality) will provide valuable information and has been highlighted as a known gap in the literature [51]. For mothers with lower milk volume, it may be important to know whether the nutritional composition of the milk, particularly total energy, differs from that of a mother with higher milk volume. Furthermore, determining the nutrient composition of human milk in New Zealand is particularly important because of unique local nutritional issues. In particular, iodine and selenium are low in the New Zealand food supply due to poor soil content [52] and are critical for infant development, as both support thyroid hormone production and regulation, and therefore brain maturation [53,54].

The aim of the Māmā and Baby Breastfeeding Study (MABBS) is to describe human milk volumes produced by a diverse sample of lactating mothers of infants aged 3 months with differing perceptions of milk supply. We aim to compare the lactating mothers’ perceptions of milk supply with the volume of milk they produce, as well as the nutrient composition of that milk.

### Primary Objective

The primary objective is to estimate the human milk intakes of fully breastfed infants aged 3 months using the gold standard DTM D_2_O technique.

### Secondary Objectives

The secondary objectives, at 3 months post partum, are to (1) estimate how human milk volumes differ between lactating mothers with differing perceptions of milk supply, (2) explore the characteristics that may predict human milk volumes produced by a diverse range of lactating mothers, (3) explore the relationship between human milk volumes produced and infant growth as a potential means of assessing human milk adequacy, (4) investigate the association between human milk composition and human milk volumes, and (5) assess how demographic characteristics might modify the relationship between perception vs actual milk supply of lactating mothers.

While the term mother is used throughout the manuscript, we acknowledge that not all lactating individuals identify as mothers. However, due to the name of the methodology used (dose-to-mother stable isotope technique) the word mother is used in this manuscript with the inclusion of all breastfeeding, chestfeeding, and human milk–feeding individuals in mind, regardless of how they identify.

## Methods

### Ethical Considerations

The study has been approved by the University of Otago Human Ethics Committee (Health) Te Pae Matatika Tangata (Haurora) Ōtākou Whakaihu Waka (H24/0225), and written informed consent will be obtained at the first appointment. The study is registered with the Australian New Zealand Clinical Trials Registry (registration number ACTRN12625000180415). Upon enrollment, participants will be assigned a unique identifying code (participant ID). To preserve confidentiality, all data collected will be recorded against the participant ID. Participants will be given a NZ $100 (US $60) grocery voucher as a thank-you for their involvement.

### Study Design

The MABBS is an observational, cross-sectional study of human milk intakes in infants aged 2 to <4 months in Dunedin, New Zealand. Participants will be included if they are: a lactating mother aged 16 years or older; living in Dunedin, New Zealand; have an infant aged 2 to <4 months at the time of their first visit; are fully breastfeeding (ie, their infant is receiving human milk only) for 2 weeks prior to recruitment; and intend to continue fully breastfeeding for the duration of the study (4 weeks).

We will exclude participants who cannot provide informed consent or complete study procedures in English, are tandem breastfeeding (simultaneously breastfeeding another child), or are breastfeeding multiples (twins or higher-order multiples). To our knowledge, the DTM technique has not been specifically validated for use in twins or tandem-fed children; therefore, the study design was based on estimating human milk intake in singleton dyads.

### Participants and Recruitment

A total of 150 mother–infant dyads will be recruited. We initially planned to target recruitment to mothers with infants aged 2 to 3 months, so that they would participate within the study age range of 3 to <4 months. This time point was selected because the prevalence of breastfeeding drops dramatically around 4 months of age in New Zealand [4], allowing estimation of human milk intake just before this decline to understand factors that may contribute to the decrease around 4 months. While some literature suggests that PIMS is most common early in the breastfeeding journey [8,55], research in New Zealand shows that PIMS is a primary reason for early breastfeeding cessation around 4 to 5 months of age [14].

Furthermore, around 3 to 4 months of age, many PIMS-related factors may contribute to this decline, including changes in infant feeding behaviors, such as an increased rate of cluster feeding (close intervals between breastfeeds), and maternal changes, such as softer breasts and less milk leakage as the body becomes more efficient at producing milk. This time point also occurs before the majority of mothers return to work in New Zealand [56] and before the recommended age for introducing complementary foods, both of which events which influence infant feeding.

However, when recruitment commenced, we found that many mothers had already begun mixed feeding before 3 months of age, potentially due to PIMS. As many of these women were from ethnic groups other than New Zealand European, the 1-month age band (3 to <4 months of age) appeared to limit the ethnic diversity of our sample. Thus, from August 29, 2025, we revised the minimum age of inclusion to 2 months rather than 3 months of age, resulting in a final inclusion period of infants aged between 2.0 and <4 months, and therefore the recruitment age range expanded to infants aged 1.5 to 2 months.

Recruitment will occur through advertisement on social media, word of mouth, and referral through health professionals (eg, midwives, Well Child Tamariki Ora providers, and lactation consultants) where possible, and by study posters in parent groups and community spaces as needed. We aim to recruit a diverse range of participants in terms of ethnicity and socioeconomic status, including approximately 20% Māori (indigenous people of New Zealand, to reflect local statistics of the region in which data collection is occurring) [57]. Some targeted recruitment (identifying and approaching groups to promote the study) may be required to achieve an ethnically diverse sample. This will occur through using networks within the maternity and health care sector as well as with Māori research colleagues.

Interested individuals will complete an online eligibility questionnaire administered through REDCap (Research Electronic Data Capture; Vanderbilt University) [58], which includes access to the information sheet about the study and the consent form to review. Once eligibility is confirmed, the potential participant will be sent an email offering them the opportunity to ask any questions and attaching the information sheet and consent form. A follow-up phone call or text will be set around 3 days later to confirm they have read the information, answer any questions about the study, obtain verbal consent, and schedule their appointments.

### Data Collection

#### Overview

Participation in the study will involve 5 contacts over 4 weeks following recruitment, as depicted in Table 1. Data collection will be conducted in the participant’s home or in the research clinic (Department of Human Nutrition, University of Otago, Dunedin, New Zealand), depending on the participant’s preference. The first visit (visit 1) will involve obtaining written consent, completion of the main questionnaire (demographic information, breastfeeding history and self-efficacy, perception of milk supply, maternal and infant sleep, maternal diet and health history, and maternal stress), obtaining anthropometric measurements (infant weight and length and maternal weight and height), obtaining baseline saliva samples from both the mother and the infant, and administration of a 30-gram dose of stable isotope (D_2_O) to the mother. The mother will be asked to take her own (mother only) at-home saliva sample 3 to 4 hours after the dose is administered (to be retrieved at visit 2). Postdose saliva samples will be taken by researchers at the second visit (visit 2), occurring 2 to 3 days after administration of the isotope, as well as at the third visit (visit 3), 6 to 7 days after the isotope, and at the fourth visit (visit 4), 13 to 14 days after isotope administration. Infant anthropometric measurements (weight and length) will be repeated at the fourth visit (visit 4). After the fourth visit (visit 4), the mother will be asked to express a full milk expression from a single breast. The final visit (visit 5) will be conducted by an International Board-Certified Lactation Consultant (IBCLC), who will take the final anthropometric measurements from the infant; conduct an observed breastfeed with a one-off, single test weighing (pre- and postfeed weights); conduct an oral assessment of the infant; and ask the mother to complete a questionnaire about dysphoric milk ejection reflex (D-MER) [59].

**Table 1:** Overview of participant contacts.

Study stage	Procedures
Recruitment: infants aged 0-2 months	Recruitment of potential participants
Screening: infants aged 6 weeks to 2 months	Eligibility questionnaire
Visit 1 (baseline): infants aged 2 to <4 months	Written consent Baseline saliva samples (mother and infant) Isotope administration (mother only) Anthropometry (mother and infant) Main questionnaire Note: the mother is asked to collect a saliva sample (mother only) 3-4 hours after the dose is administered
Visit 2: 2-3 days after baseline	Follow-up (postdose) saliva samples (mother and infant) Collect postdose saliva sample, that was self-collected by the mother after visit 1 (mother only)
Visit 3: 6-7 days after baseline	Follow-up (postdose) saliva samples (mother and infant)
Visit 4: 13-14 days after baseline	Final (postdose) saliva samples (mother and infant) Anthropometry (infant only)
Human milk collection: approximately 3 days after visit 4	Collection of 12 mL subsample of full milk expression from single breast
Visit 5: 2 weeks after visit 4	Anthropometry (infant only) Single test-weigh = weight before and after a breastfeed (infant) Breastfeeding assessment Oral assessment D-MER^a^ questionnaire

^a^D-MER: dysphoric milk ejection reflex.

#### Demographic Data

Self-reported maternal and mother-reported child ethnicity will be collected as part of the eligibility questionnaire, using standard New Zealand census questions [60]. Area-based household socioeconomic deprivation (New Zealand Index of Deprivation 2023) will be determined using the participant’s postal address [61]. New Zealand Index of Deprivation 2023 combines 9 variables from the 2023 New Zealand national census to determine a socioeconomic deprivation score for each statistical area (a geographic area defined by Statistics New Zealand containing approximately 100 to 200 people). The deprivation score estimates the relative material and social deprivation for the area where the participant lives, where decile 1 represents areas with the least deprivation and decile 10 represents areas with the most deprivation. Maternal education, maternal work status, childcare use, and the number of children and adults living in the household will be collected in the main questionnaire administered at the first visit.

#### Maternal Perception of Milk Supply

Perception of milk supply will be assessed in the main questionnaire using 2 approaches. First, using the Perceived Infant Breastfeeding Satiety Subscale of the Hill and Humenick Lactation Scale [15] in the main questionnaire, which is a validated scale designed to determine PIMS. The Perceived Infant Breastfeeding Satiety Subscale is a 5-question subscale with responses recorded using a 7-point Likert scale (1=strongly disagree to 7=strongly agree). Higher scores represent higher perceived milk supply.

Second, a single question developed by the research team will be administered to assess maternal perception of milk supply: “How do you currently feel about the amount of breast milk you make for your baby/pēpi? (Think about over the past week.)” with answers from a 5-point Likert scale (1=not enough milk, 2=sometimes not enough milk, 3=enough milk, 4=sometimes too much milk, and 5=too much milk). This continuous variable will be dichotomized to create a variable for comparing low (insufficient) perceived milk supply and high (oversupply) perceived milk supply, combining 1=not enough milk and 2=sometimes not enough milk to define low (insufficient), and 4=sometimes too much milk and 5=too much milk to define high (oversupply), relative to 3=sufficient supply.

Participants will also be asked whether they have had any prior concerns about their milk supply and, if so, when these concerns occurred, what caused these concerns, and whether the participant did anything to address these concerns. Additionally, at visits 1 to 4, participants will be asked whether there have been any new concerns about low milk supply to determine whether their perception changes over the course of the DTM period.

#### DTM Stable Isotope Technique

##### Human Milk Volume

Human milk volume will be determined using the gold-standard stable isotope DTM technique [24,32,33]. Baseline saliva samples (approximately 2 mL) will be collected from the mother and infant participants at the first visit. Participants will be asked to ensure that neither they nor their baby has consumed any food or drink for 30 minutes prior to saliva sample collection (to prevent contamination of the sample with any food or drink residue).

After ensuring appropriate sanitization, the researcher, using gloved hands, will place a dental cotton roll (Premium Dental Cotton Rolls, Henry Schein) into the mother’s mouth to soak up saliva, without the cotton roll being touched by the mother’s hands. Once sodden, the cotton roll will be placed into a 10 mL syringe and squeezed into a labeled tube (1.5 mL; screw top) until at least 1 mL of saliva is extracted. This will be repeated to fill a second tube as a spare, in case of any measurement errors or spills during analysis.

For the infant’s saliva sample, the same process will be followed; however, the researcher will hold 1 cotton roll and move it around the lower left and right sides of the infant’s mouth and under the tongue until sodden. Filled sample and spare tubes will be placed into separate “mother” and “baby” labeled plastic ziplock bags and into a cooler box with an ice pack before being transferred into a –20 °C freezer until analysis.

After the baseline samples have been taken, an accurately measured dose of D_2_O (Aldrich, 99.8 atom% deuterium) will be administered to the mother participant as a drink of water through a straw. The dose amount will be 30 g measured to the nearest 0.01 g (ie, doses will range from a minimum of 30 g to a maximum of 30.09 g). To ensure the full dose is consumed by the participant, the dose bottle will be filled twice with 50 mL of regular drinking water, inverted, and consumed by the mother through the same straw.

The elimination of D_2_O from the mother and transfer to the infant will be traced over the 14-day collection period through analysis of deuterium enrichment of the saliva samples. Postdose (D_2_O) saliva samples will be collected using the same methods described above from the mother and infant at 3 time points: 2 to 3 days postdose, 6 to 7 days postdose, and 13 to 14 days postdose. The reduced sampling protocol from the original International Atomic Energy Agency (IAEA) protocol will be used [24], which has shown high sensitivity and specificity [33].

Infant morbidity will be assessed at visits 3 and 4 with a one-off question: “Has baby been unwell in the last week?” to determine whether any illness occurred over the DTM period.

Any participants expressing breast milk will be asked to weigh and record the amount of milk expressed and indicate whether this was fed back to the infant, either fully or partially, during the 14-day DTM period. An expression log and electronic kitchen scale (Salter, model 1023) will be provided to the participant for recording purposes. Participants will be asked not to feed the infant any expressed milk that had been stored prior to the consumption of D_2_O, as this cannot be tracked; therefore, infants will only consume expressed milk from the 14-day DTM period. Specific expression storage bags will be provided to participants to ensure accurate tracking of milk expressed during the DTM period and to minimize any confusion with milk expressed and stored outside of this time.

##### Maternal Fat Mass and Fat-Free Mass

Maternal body composition will be measured through the DTM technique, providing calculated fat mass and fat-free mass [24]. Participants will be asked to collect their own saliva sample 4 hours (or at least between 3 and 4 hours) after administration of the stable isotope, using the same methods used by the researcher at visit 1. They will be provided with equipment and instructions and asked to store the sample in their refrigerator until it is collected by a researcher at the second visit.

#### Human Milk Sample Collection

At the end of the DTM assessment period, a subsample of a complete human milk sample (full expression) will be collected from the participant in their home. At visit 4, participants will either be provided with a sterile breast pump (manual or electric) and collection bottle or given the option of using their personal breast pump, depending on preference. For those using their own pump, a microwave steam sterilizer bag (Medela or New Beginnings) will be provided so that the participant can sterilize the pump parts prior to expression, to minimize contamination.

Participants will be asked to, prior to expression, to clean their hands and the breast area with demineralized water and a cleaning gauze as provided, in order to remove any trace elements from creams that may have been applied to the hands or nipple area (eg, barrier creams, which commonly contain zinc). Participants will be asked to express a full milk sample from 1 breast in the morning (before 12 PM), at least 2 hours after the last feeding or pumping session on that breast, to standardize the collection period.

This method of human milk collection has been shown to be representative of the milk that an infant consumes, and by standardizing the collection period for all participants, the samples will be appropriate for comparisons between participants at the same time point post partum [62]. Participants will be asked to gently invert their full milk sample 3 times immediately after collection and then pour a 12-mL subsample into a collection container provided by the study that has been labeled with the participant ID and to indicate on the container the volume collected. The 12-mL amount was chosen to balance analytical needs (this is the volume required for the planned analyses) with sensitivity to mothers who may have concerns about their milk supply (their infant can be fed the remainder of the expressed milk).

#### Breastfeeding Observation

A one-off breastfeeding will be observed in the final visit (visit 5) by an IBCLC. The IBCLC will use the Bristol Breastfeeding Assessment Tool [63] to assess adequate latch and transfer of milk by the infant. This tool has 4 main elements, scoring a breastfeeding on positioning, sucking, swallowing, and attachment. A score of 0 (poor feeding elements) to 8 (good feeding elements) will be recorded by the IBCLC on the standardized Bristol Breastfeeding Assessment Tool form [63].

#### Test Weighing

During the breastfeeding observation by the IBCLC, a single test weighing of human milk transfer will be taken using an electronic baby scale (Seca model 334 or Marsden model M-300). Single test weighing, the process of weighing the infant before and after a single breastfeed, is often used clinically by health care professionals working with breastfeeding dyads to assess adequate milk transfer. The volume of milk transferred in the single test weighing will be compared with the human milk volume estimated by the DTM technique to investigate whether a one-off test weighing is indicative of actual human milk volume over a 2-week period.

#### Anthropometry

Infant weight and length will be measured using an electronic baby scale (Seca model 334) and a 99-cm measuring mat (Seca model SE210). Infants will be weighed naked with only a dry nappy of known weight. Maternal weight (minimal clothing, no shoes) will be measured using an electronic scale (Tanita model HD-351), and height will be measured using a portable stadiometer (Wedderburn model WMHM200P or Seca model 213), depending on whether the visit is at the participants’ home or in the clinic. Measurements will be conducted by trained research staff in duplicate, following the World Health Organization (WHO) anthropometric protocol [64]. A third measurement will be taken if the difference between the first and second measurements exceeds the maximum allowable difference (0.1 kg and 0.5 kg for infant and maternal weight, respectively, and 0.7 cm for length and height).

At visit 5 (the final visit), photographs will be taken of anthropometric measurements collected during Well Child Tamariki Ora visits. These will be entered into REDCap. Well Child Tamariki Ora visits are free health assessments that are offered to all New Zealand families and conducted between ages 6 weeks and 5 years [65]. These anthropometric data will be used alongside those directly measured in the study to assess infant growth trajectory over time, which will be compared with the WHO Child Growth Standards [66]. Infant weight and length data will be used to calculate BMI and determine BMI-for-age *z* score using sex-specific reference data from the WHO Child Growth Standards [66].

#### Perception of Infant and Maternal Sleep

Infant sleep will be assessed using questions from a New Zealand sleep study [67], modified to include questions about infant sleep location and changes to infant sleep patterns. Participants will be asked a question about their own sleep quality and sleep quantity, developed by the research team, in the main questionnaire to assess maternal sleep perception.

#### Potential Predictors of Human Milk Intake

##### Overview

Human milk intake is complex and can be influenced by a range of different factors. A selection of potential maternal and infant factors that may influence human milk intakes has been identified through literature searches and advice from breastfeeding experts. These are summarized in Table 2.

**Table 2:** Potential predictors (maternal and infant) of human milk intakes.

Factors that potentially predict human milk intakes		Explanation of potential impact or hypothesis	References
**Maternal factors**			
	Breastfeeding self-efficacy	Mothers who have greater self-efficacy may have infants with higher intake.	Khresheh and Ahmad [68]
	Breast or chest trauma	Mothers who have experienced breast or chest trauma may have infants with lower intake.	Kraut et al [69] Haifeng et al [70] Stopenski et al [71] Leal et al [72] Holmgren et al [73]
	Cannabis	Mothers who use cannabis may have infants with lower intake.	Castro-Navarro et al [46]
	D-MER^a^	Mothers who experience D-MER (sudden, intense negative emotions that occur immediately before and during milk let-down) may have infants with lower intake.	Ureño et al [74]
	Early return of menses	Mothers who have had return of menses may have infants with lower intake.	Spencer et al [49]
	Education	Mothers who are more educated may have infants with higher intake.	Bandara et al [39] Haszard et al [31]
	Employment (current)	Mothers who are in current employment may have infants with lower intake.	Haszard et al [31]
	Following specific diet	Mothers who are consuming specific dietary patterns (eg, low fat, dairy-free, and vegetarian) may have infants with differing intake.	Spencer et al [49]
	Galactagogues or antigalactagogues	Mothers who are consuming galactagogues (a substance that is thought to increase milk volume) may have infants with higher intake, and mothers who are consuming antigalactagogues may have infants with lower intake.	Brodribb et al [75] Johnson et al [76]
	Hormonal birth control	Mothers who are taking hormonal birth control may have infants with lower intake.	Berens et al [77]
	Maternal BMI	Mothers with higher BMI may have infants with lower intake.	Hilson et al [40] Haszard et al [31]
	Maternal fat mass	Mothers who have higher fat mass may have infants with lower intake.	Diana et al [78]
	Parity	Mothers who have previously given birth may have infants with lower intake.	Whitehead et al [41]
	Previous breastfeeding experience	Mothers with previous breastfeeding success may have infants with higher intake, while those who did not have success previously may have infants with lower intake.	Huang et al [79]
	Maternal morbidities	Mothers who have medical conditions may have infants with lower intake.	Spencer et al [49]
	Maternal sleep	Hypothesis that maternal sleep may impact infant milk intake.	N/A^b^
	Maternal use of medications	Mothers on certain medications may have infants with differing intake.	Farah et al [48]
	Mental health	Mothers who experience challenges with mental health may have infants with lower intake.	Spencer et al [49]
	Pain while breastfeeding	Mothers who experience pain while breastfeeding may have infants with lower intake.	McGuire [80]
	Smoking or nicotine	Mothers who use nicotine may have infants with lower intake.	Napierala et al [47]
	Stress	Mothers with higher levels of stress may have infants with lower intake.	Nagel et al [50]
**Infant factors**			
	Age	It is known that infant intake varies over the course of lactation.	Bandara et al [39] da Costa et al [23] Whitehead et al [41] Brown et al [42] Haszard et al [31]
	Body weight	Infants with smaller body weight may have lower intake.	Bandara et al [39] Daniels et al [22] Agne-Djigo et al [43] Haisma 2003 [34] Haszard et al [31]
	Demand vs schedule feeding	Infants fed on demand rather than by schedule may have higher intake.	Dewey et al [44] Haszard et al [31]
	Growth trajectory	Infants with greater growth velocity may have higher intake.	Olga et al [36]
	Household temperature and humidity	Higher household temperature and humidity may be associated with higher intake.	Baby et al [81]
	Number of breast feeds (average per day)	Infants feeding more often may have higher intake.	Brown et al [42] Haszard et al [31]
	Sex	Male infants may have higher intake than female infants.	da Costa et al. 2010 [23]
	Spilling (average quantity)	Hypothesis that intake may differ in infants who spill.	N/A
	Term, preterm, or early term	Infants born early may have lower intake.	Lapillonne et al [82] Altuntas et al [83] Boies and Vaucher [84]

^a^D-MER: dysphoric milk ejection reflex.

^b^N/A: not applicable.

##### Breastfeeding Self-Efficacy

Maternal breastfeeding self-efficacy will be assessed using the Breastfeeding Self-Efficacy Scale–Short Form [85] in the main questionnaire. This is a validated 14-item questionnaire with a 5-point Likert scale (1=not confident to 5=very confident) to determine the participant’s confidence in their ability to breastfeed. Total scores range from 14 to 70, with higher scores indicating higher level of breastfeeding self-confidence.

##### Health History

Maternal and infant health history will be collected in the main questionnaire. Questions about birth and breastfeeding experience, breastfeeding support and practices, infant gestational age, parity, current medications or medical conditions, use of supplements, maternal dietary pattern and appetite, breast surgery, and return of menses will be included to determine their potential impact on human milk intake.

The main questionnaire will also include questions about breastfeeding practices, such as the average number of breastfeeds in 24 hours, whether participants are breastfeeding on demand or on a schedule, how much the infant spills, and questions on previous breastfeeding experience. Maternal stress will be assessed using the Global Measure of Perceived Stress Questionnaire [86].

Questions about nipple or breast trauma will be asked at visit 1 and repeated at subsequent visits (visits 2 to 4) to determine whether any changes occurred during the study period. Breastfeeding pain and maternal experience with D-MER will be assessed at visit 5 (final) with the IBCLC.

Breastfeeding pain will be the mother’s self-reported usual pain during a breastfeed on a scale of 0 (no pain) to 10 (worst pain). If pain is reported as greater than 0, follow-up questions will be asked about the type of pain, timing, frequency, and its impact on breastfeeding. This will be assessed separately from the questions around nipple or breast trauma, as those focus on damage and injury rather than general pain. Presence of D-MER will be assessed using the validated D-MER questionnaire [59]. Those who are experiencing symptoms of pain or D-MER will be offered further support by the IBCLC based on clinical judgement.

##### Nicotine and Cannabis Use

Questions about nicotine use will be assessed in the main questionnaire. Due to the rising rates of electronic cigarette (“vape”) use in New Zealand [87] and internationally [88], as well as evidence that use could be negatively associated with breastfeeding duration [89], questions on cigarette and electronic cigarette use will both be included. The number of cigarettes per day, as well as the use, nicotine level, and flavor of electronic cigarettes, will be assessed via questionnaire.

Cannabis use will be assessed in the main questionnaire. As cannabis is illegal in New Zealand, care will be taken when asking these questions to ensure that confidentiality is maintained and participants feel safe responding. Participants will be reminded that all responses are confidential and no identifying information will be kept alongside their responses. The questions about cannabis will be optional, allowing participants to leave this section blank if they feel more comfortable. Cannabis use will be assessed via questions used previously [90], modified to include questions about use after birth, along with use during pregnancy.

##### Household Temperature and Humidity

To explore the impact of potential heat stress on infant milk intake, household temperature and humidity will be measured using a digital hygrometer (Niome) with a temperature accuracy of ±1 °C and humidity accuracy of ±5%. For participants who choose to have their visits in the home that the infant is most commonly in, the digital hygrometer will be placed in the main room of the home where the visit takes place. The household temperature and humidity will be recorded after 15 to 20 minutes, which gives the hygrometer enough time to obtain an accurate reading of the room.

### Laboratory Analyses

#### Human Milk Volume

The deuterium enrichment in saliva samples will be determined by Fourier transform infrared spectrometry (Thermo Fisher Nicolet Summit, Thermo Fisher Scientific) with a liquid accessory attachment (SPECAC Pearl and CaF_2_ 100-µm pathlength top and bottom window plates). Enrichment will be determined using a modified method described by the IAEA [24]. Previously frozen saliva samples will be thawed at room temperature for 30 minutes, inverted 4 times, and centrifuged at 1000 × g for 10 minutes.

A trained researcher will pipette 45 µL of sample (baseline or postdose) onto the cell window, close the top cell window, and measure the absorption spectrum based on settings described in the IAEA protocol [24]. Each sample will be measured in duplicate unless the difference between the 2 sample measurements is greater than 3 mg/kg, in which case a third or fourth measure will be taken until 2 measurements are less than 3 mg/kg apart. The mean of the 2 closest measurements (within 3 mg/kg) will be the calculated D_2_O at each time point.

Following the determination of D_2_O enrichment, the calculation of human milk volume, infant nonmilk water intake, and classification of exclusive breastfeeding will be determined using a validated method [32,33]. The mother’s measured height and weight and the infant’s measured length and weight (collection described above) will be used in the calculation of human milk volume.

#### Human Milk Composition

##### Macronutrients

Human milk samples will be collected from the participant on the same day of expression. Samples will be carefully mixed and aliquoted into a 10-mL collection tube and frozen within 48 hours of collection, at –20 °C. Frozen samples will be defrosted at room temperature and analyzed within 2 hours. Once samples are defrosted, they will be placed into a heating bath where they will sit for approximately 20 minutes to warm to 40 °C. When the samples have reached 40 °C, they will be mixed by inversion and homogenized using an ultrasonic processor (Miris Ultrasonic Processor).

Macronutrient composition will be analyzed using a human milk analyzer (HMA, Miris). A total of 3 mL of sample will be slowly syringed into the HMA and repeated in triplicate (3 analyzes per sample) to ensure accuracy of the reading.

The HMA uses a combination of midinfrared transmission spectroscopy principles to measure concentrations of fat, carbohydrate (lactose and oligosaccharides), and both crude and true protein in human milk, as well as providing calculated values for total solids and energy. The HMA software uses internal calibration based on chemical standard methods validated for analysis of human milk. The Röse-Gottlieb method is used for total fat [91], and Kjeldahl [92] for crude protein. True protein is crude protein multiplied by 0.8 to correct for nonprotein nitrogen. The total carbohydrate content is calculated as total solids minus fat, protein, and ash [93]. Total solids are measured by drying oven [94].

##### Minerals and Trace Elements

Minerals and trace elements will be determined following methodology used previously [22]. Specifically, minerals (sodium, magnesium, phosphorus, potassium, and calcium) and trace elements (iron, copper, zinc, selenium, and iodine) will be analyzed in the Centre for Trace Element Analysis, Department of Chemistry, University of Otago, New Zealand, by inductively coupled plasma mass spectrophotometry (Agilent 7900; Agilent Technologies). Briefly, 10 mL of quartz sub-boiling 14 N nitric acid and 1 mL of 30% hydrogen peroxide will be added to approximately 1 g of the human milk sample. Samples will be microwave-digested at 200 °C for 15 minutes and dried down to approximately 1 mL. The final sample will be diluted to 25 mL with 2% aqueous nitric acid. Precision and accuracy will be checked using in-house pooled samples and multielement reference standards (SRM 1549a, whole milk powder) from the National Institute of Standards and Technology.

### Data Management

Upon enrollment, participants will be assigned a unique identifying code (participant ID). To preserve confidentiality, during data collection all data will be recorded against the participant ID. The key linking the participant ID with the participant’s name and address will be kept secure. Although it will need to be accessible to research staff so they can attend home visits (name and address) and collect data (participant ID), research staff will only have access to health information when it is essential for specific study-related tasks.

Responses to online questionnaires will be linked to participant ID numbers. Participant IDs will also be written on biological sample tubes and recording sheets. All study data will be stored in the secure REDCap database [58]. In the unlikely event that data cannot be entered directly into the REDCap database, they will be entered on a hard-copy datasheet and later transferred into REDCap. All paper copies will be stored in a locked office.

Data will be collected following rigorous protocols and cleaned according to standard data entry procedures. Quality control visits (observations of research staff conducting visits) will be conducted by senior research staff to ensure that staff follow data collection protocols consistently. Data quality checks will be run on all entered data to check for accuracy, consistency, and completeness. The results database will be stored on research team members’ computers, all of which are password protected.

### Statistical Analysis

#### Sample Size

We anticipate a dropout rate, including incomplete data, of 20% based on recruitment and completion data from a similar recent study [95]. Therefore, we aim to recruit 150 mother–infant dyads, which is expected to result in approximately 120 with complete data. With 120 participants, mean human milk intake can be estimated to a 95% CI of SD 0.18. If the distribution of intakes is similar to previous data, this corresponds to around ±27 mL/day (ie, 3% of an estimated intake of 787 mL/day at 3 months [22]). A final sample of 120 will provide a robust sample size to estimate the correlation between human milk intake and PIMS, as well as to estimate differences between groups [96].

#### Data Analysis Plan

Human milk intakes of infants aged 3 months will be described. Perception of milk supply will be collected as a continuous scale and dichotomized to create a variable for comparing low (insufficient) perceived milk supply and high (oversupply) perceived milk supply. Regression models will be used to assess how maternal perception of human milk supply (categorical independent variable) is associated with actual human milk volumes produced by lactating participants at 3 months post partum (dependent variable). Mean differences and 95% CIs will be estimated. Similarly, the relationships between predetermined potential predictors (Table 1) of human milk intakes and actual milk intakes will be described using regression modeling. Residuals of regression models will be plotted to assess homoscedasticity and normality. Results will be presented for descriptive purposes; therefore, no adjustment for confounders will be made. However, moderation by demographic characteristics will be investigated descriptively by presenting stratified results. Data will be illustrated graphically where appropriate.

To understand whether certain factors predict breastfeeding problems, the breastfeeding assessment tool will be used. These data will be analyzed using prediction modeling techniques, including a combination of linear regression models (reporting *R*^2^), and least absolute shrinkage and selection operator regression, to understand which factors might combine to predict breastfeeding problems. In addition, if there are any modifiable factors indicated to cause breastfeeding problems, a causal inference approach to analysis will be used to determine which confounders to include in the model, including the development of a directed acyclic graph.

BMI and weight velocity between birth and age up to 5 months will be calculated from the Well Child Tamariki Ora data collected. An initial investigation will be conducted into whether infant milk intakes and infant growth over time (birth to age up to 5 months) can be used to assess human milk inadequacy (actual low milk volume) using regression models.

Statistical analyses will be conducted under the guidance of a biostatistician using Stata (version 18; StataCorp LLC) software.

## Results

This study was funded in April 2023 by the Health Research Council of New Zealand (grant 23/461). Recruitment for this study began in February 2025 and is currently underway. As of January 17, 2026, a total of 91 participants have been enrolled. Recruitment is anticipated to conclude in June 2026, with analysis expected to be completed by February 2027. Final results are anticipated to be disseminated in late 2027 following completion of data analysis.

## Discussion

This study will explore the relationship between maternal perception and actual milk quantity and quality, providing significant implications for improving breastfeeding support. If PIMS is linked to specific physiological characteristics of human milk, such as lower volume or suboptimal nutrient concentrations, this could provide a biological basis for potentially individualized interventions to support the continuation of breastfeeding. Alternatively, if perception is not linked to measurable differences, this would reinforce the need for better psychosocial support and education to sustain breastfeeding, or the development of tools that can be used to assess milk supply and reassure mothers that their supply is sufficient. Because perception is a modifiable factor, identifying its relationship to human milk volume and composition offers a critical opportunity to intervene early, support breastfeeding continuation, and improve infant and maternal health outcomes.

To the best of our knowledge, there is currently no published research using the DTM technique to investigate whether PIMS is related to actual milk insufficiency, nor has the association with nutrient composition been investigated, a gap in the literature as highlighted by the 2023 Lancet Series on Breastfeeding [51]. While 1 study has investigated the relationship between perceived and actual milk insufficiency [20], it was restricted to first-time mothers and used test-weighing methods to estimate infant milk intake, both of which inherently limit the interpretation of the findings.

The main strength of MABBS is the use of the DTM stable isotope technique for estimating human milk volume in relation to mothers’ perception of milk supply. This methodology is considered to be the gold standard, allowing the most accurate reporting of infant milk intake [33]. This is particularly important given our large sample size (at least 120 participants), which allows us to determine daily infant milk intake to an accuracy of ±27 mL/day. Finally, we aim to recruit a diverse population with good ethnic representation to ensure that the outcomes are relevant to all people living in New Zealand.

This study also has some limitations. A narrow age range of participants (those with babies aged 2 to <4 months) was required because of the variation in milk intake at different stages of lactation. While this is necessary for the methodology of this study, it may impact our ability to capture participants with a wide range of perception of milk supply. Furthermore, while the research team aim to make the results relevant to the New Zealand population, due to differences in populations, the findings of this study may not be generalizable to people in other countries outside New Zealand.

We will disseminate the results of this study through multiple channels. For academic dissemination, the results will be published in open-access and high impact journals, as well as presented at relevant conferences. However, it is of high importance to members of the research team to ensure that results are also useful to inform practice at a clinical level. With help from the advisory group and experts in this space, results will be shared with those working with breastfeeding families to help shape clinical practice. This may include webinars or resources specifically designed for health professionals to inform personalized intervention strategies for breastfeeding parents.

## Appendix

Multimedia Appendix 1 Peer review report from HRC.

## Abbreviations

D2O: deuterium oxide
D-MER: dysphoric milk ejection reflex
DTM: dose-to-mother
HMA: human milk analyzer
IAEA: International Atomic Energy Agency
IBCLC: international board-certified lactation consultant
MABBS: Māmā and Baby Breastfeeding Study
PIMS: perceived insufficient milk supply
REDCap: Research Electronic Data Capture
WHO: World Health Organization

## Data Availability

The datasets generated or analyzed during this study are not publicly available due to ethical restrictions related to the consent provided by participants but are available from the corresponding author on reasonable request.
