# Supplementary material for: Maternal Perception vs Actual Breast Milk Supply: Protocol for an Observational Cross-Sectional Study
Source: JMIR Res Protoc. 2026 Mar 31;15:e84776. doi: 10.2196/84776 (PMC13080296; doi:10.2196/84776)
Supplement: Multimedia Appendix 1 [file resprot_v15i1e84776_app1.pdf]

Emerging Researcher First Grant Review Summary

**HRC reference #**	23/461	**Applicant surname**	Daniels
**Title of research**	Maternal perception vs actual breast milk supply: is there a difference?		
**Host**	University of Otago		

With regard to the criteria for assessing and scoring research proposals:

1. The assessing committee noted the following key strengths of the application (brief bullet points)

- The committee noted that the applicant was a strong candidate with an impressive publication record, good student supervision experience and was well supported by organisation and research team.
- The application provided a strong rationale for undertaking this important area of research.

2. The assessing committee noted the following aspects that could be improved and/or considered further (brief bullet points)

- The committee felt the application needed more description of the 150 participants, particularly regarding any confounding factors such as mother's ability to breastfeed and factors that could prevent breastfeeding.
- Similarly, the committee queried if the proposed milk volume needed adjustment for varying maternal and baby weights?
- The committee would have liked to have seen more clarity around the specific form of mātauranga Māori applied in the study. This could provide a different perspective to breastfeeding than Western paradigms.

3. Other Comments/suggestions (brief bullet points)

- The project could have been enriched with dedicated funding for a named Māori investigator.
